# Effective Linkages of Continuum of Care for Improving Neonatal, Perinatal, and Maternal Mortality: A Systematic Review and Meta-Analysis

**DOI:** 10.1371/journal.pone.0139288

**Published:** 2015-09-30

**Authors:** Kimiyo Kikuchi, Evelyn Korkor Ansah, Sumiyo Okawa, Yeetey Enuameh, Junko Yasuoka, Keiko Nanishi, Akira Shibanuma, Margaret Gyapong, Seth Owusu-Agyei, Abraham Rexford Oduro, Gloria Quansah Asare, Abraham Hodgson, Masamine Jimba, Ghana EMBRACE Implementation Research Project Team

**Affiliations:** 1 Department of Community and Global Health, Graduate School of Medicine, the University of Tokyo, 7-3-1 Hongo, Bunkyo-ku, Tokyo, 113–0033, Tokyo, Japan; 2 Research and Development Division, Ghana Health Service, Accra, Ghana; 3 Dodowa Health Research Centre, Dodowa, Greater Accra, Ghana; 4 Kintampo Health Research Centre, Kintampo, Brong-Ahafo, Ghana; 5 Navrongo Health Research Centre, Navrongo, Upper-East, Ghana; 6 Ghana Health Service, Ministries, Accra, Ghana; Centre Hospitalier Universitaire Vaudois, FRANCE

## Abstract

**Background:**

Continuum of care has the potential to improve maternal, newborn, and child health (MNCH) by ensuring care for mothers and children. Continuum of care in MNCH is widely accepted as comprising sequential time (from pre-pregnancy to motherhood and childhood) and space dimensions (from community-family care to clinical care). However, it is unclear which linkages of care could have a greater effect on MNCH outcomes. The objective of the present study is to assess the effectiveness of different continuum of care linkages for reducing neonatal, perinatal, and maternal mortality in low- and middle-income countries.

**Methods:**

We searched for randomized and quasi-randomized controlled trials that addressed two or more linkages of continuum of care and attempted to increase mothers’ uptake of antenatal care, skilled birth attendance, and postnatal care. The outcome variables were neonatal, perinatal, and maternal mortality.

**Results:**

Out of the 7,142 retrieved articles, we selected 19 as eligible for the final analysis. Of these studies, 13 used packages of intervention that linked antenatal care, skilled birth attendance, and postnatal care. One study each used packages that linked antenatal care and skilled birth attendance or skilled birth attendance and postnatal care. Four studies used an intervention package that linked antenatal care and postnatal care. Among the packages that linked antenatal care, skilled birth attendance, and postnatal care, a significant reduction was observed in combined neonatal, perinatal, and maternal mortality risks (RR 0.83; 95% CI 0.77 to 0.89, I^2^ 79%). Furthermore, this linkage reduced combined neonatal, perinatal, and maternal mortality when integrating the continuum of care space dimension (RR 0.85; 95% CI 0.77 to 0.93, I^2^ 81%).

**Conclusions:**

Our review suggests that continuous uptake of antenatal care, skilled birth attendance, and postnatal care is necessary to improve MNCH outcomes in low- and middle-income countries. The review was conclusive for the reduction of neonatal and perinatal deaths. Although maternal deaths were not significantly reduced, composite measures of all mortality were. Thus, the evidence is sufficient to scale up this intervention package for the improvement of MNCH outcomes.

## Introduction

The Millennium Development Goals (MDGs) call for reducing global maternal and child mortality by 2015. However, worldwide, approximately 289,000 women still died in 2013 due to complications of pregnancy and childbirth [1, 2]. About 99% of such deaths occurred in low-and middle-income countries (LMICs) [1]. Furthermore, the total number of under-five children’s deaths was 6.6 million in 2013, of which 80% occurred within 48 hours postpartum [1]. Only one-third of the 68 Countdown Countries are on track to achieve MDG4 (reduce child mortality), and the average annual rate of decline is far below that needed for MDG5 (improve maternal health) [1].

To improve maternal, neonatal, and child health (MNCH), various interventions have been recommended. However, most of the over 190 MNCH studies addressed only a single intervention [3–8]—few of them looked at how these interventions could be combined. Moreover, MNCH care programs are often vertical in nature; because newborn health is largely dependent on maternal health, care for both the mother and newborns should be provided in a continuous manner [9]. Indeed, around 20% of diseases among infants can be attributed to the mother’s poor health status, malnutrition, and inadequate care during the perinatal period [10]. For instance, maternal treatment of infectious diseases such as HIV and malaria influences infants’ health [11, 12].

The continuum of care has been well known as a potential means of improving MNCH through integrated service delivery [13–15]. Recent MNCH studies have begun to focus more on interventions to strengthen continuum of care [16, 17], although researchers have yet to establish a clear definition of continuum of care [3, 14]. In MNCH, continuum of care has two core dimensions: sequential time and space [3, 14, 15, 18]. The time dimension includes service delivery from pre-pregnancy to childhood, while the space dimension includes community–family care, outreach–outpatient care, and clinical care [19]. Previous studies estimated that up to 67% of neonatal deaths could be prevented by improving the coverage of common MNCH care to 90% of reproductive age women [7, 20]. However, as resources are limited, providing all possible MNCH services is unrealistic, particularly in LMICs. For instance, about 0.68–1.32 billion USD was required for providing a total continuum of care package from antenatal care to postnatal care to avert 38–67% of neonatal deaths in Sub-Saharan Africa [20]. As such, identifying the most effective MNCH package must be prioritized in health policies to ensure more effective use of continuum of care.

The frequency and timing of care delivery are important considerations in defining continuum of care. The recommended standards of MNCH care are as follows: (1) four antenatal care visits [21–24]; (2) delivery assisted by skilled birth attendants [25–27]; and (3) postnatal care within 48 hours [28–32], (4) at seven days [31, 33–37], and (5) at six weeks [31]. Regarding antenatal care, fewer visits have often been the standard in LMIC trials [24]. Compared to five antenatal care visits, fewer than five antenatal care visits does not significantly increase maternal and neonatal mortality [21, 38], which led the World Health Organization (WHO) to define four antenatal care visits as the standard in their guidelines [39]. However, both the frequency and timing of antenatal care are inconsistently applied in different MNCH interventions [40].

Postnatal care frequency and timing are also inconsistent, possibly due to changes in global postnatal care recommendations. The WHO’s practical guidelines from 1998 encourage mothers with babies to access postnatal care services at “six hours, six days, six weeks, and six months” [41]. This formula targeted “crucial” moments of need for the mother and baby, though rigorous evidence was absent. Since then, different frequencies and timings have been recommended: in 2006, the timings were within the first week, preferably 2–3 days after delivery, and 4–6 weeks [42], and in 2009, within the first 24 hours after birth, on day three, and on day seven, if necessary [30]. This was based on evidence indicating high maternal deaths on the first and second days after birth [43]. Additionally, almost 40% of under-five deaths occur within the first 28 days of life [30, 44], and three-quarters of neonatal deaths occur during the first week of life, with 25–45% in the first 24 hours [37]. Thus, we need to examine whether these standards for frequency and timing of care delivery should be considered in defining the continuum of care.

Although the expectation for continuum of care effectiveness is high and interventions have been planned, we found no prior review article or review protocol in this area. Comprehending the effectiveness of continuum of care linkages will encourage further opportunities for intervening in MNCH care and guide in tailoring interventions. Thus, in this systematic review and meta-analysis, we quantitatively synthesized evidence proposing effective linkages of continuum of care. Our focus was which linkages reduce neonatal, perinatal, or maternal mortality in LMICs in the continuum of care time and space dimension stages.

## Materials and Methods

We conducted a systematic review according to the guidelines of the Cochrane Collaboration [45], and followed the four phases indicated in the Preferred Reporting Items for Systematic Reviews and Meta-Analyses (PRISMA) Statement [46]. The PRISMA statement for our systematic review is available in S1 Table. We registered the protocol for this systematic review at the PROSPERO International prospective register of systematic reviews on October 25, 2013, and updated it on January 28, 2015. The registration number for this review is CRD42013006089. It is available at http://www.crd.york.ac.uk/PROSPERO (S1 Text).

### Study Inclusion Criteria

We included only randomized and quasi-randomized controlled trials in this systematic review, including both individual and cluster-randomized studies. Regarding publication types, we included only peer-reviewed journals and reports from international organizations. We considered papers of all languages, so long as they had English abstracts. Furthermore, we included only articles set in LMICs as defined by the World Bank [47], and excluded non-randomized studies and non-intervention studies such as case series, case reports, and qualitative studies, as well as studies conducted in high-income countries.

### Participants

We included women and neonates residing in LMICs. Women had to be in the pregnancy, intrapartum, or postpartum period. We excluded studies that included specific subpopulations that would have made it difficult to generalize the results to a wider population, such as HIV-positive women and groups at high risk for pregnancy complications.

### Interventions and Controls

Interventions comprised packaged care/services that addressed different continuum of care time and space stages. According to the WHO and the Partnership for Maternal, Newborn and Child Health, the time dimension of continuum of care starts from pre-pregnancy and progresses to childhood and motherhood [14, 48–50]. However, given that we focused on neonatal, perinatal, and maternal mortality in the review, the target periods were only the pregnancy, birth, and postnatal periods of mothers and neonates. The space dimension of continuum of care comprises three care stages—community–family care, outpatient–outreach care, and clinic care [3]. The community–family care interventions included home or community-based care practices addressing mothers and neonates’ healthy behaviors. The outpatient–outreach care included population-oriented services that health facilities or mobile services delivered routinely or periodically according to defined schedules. The clinical care interventions included individual-oriented care through facility-based care at primary and referral levels.

We defined the control groups as those who received standard care. In this study, standard care was defined as that provided in health facilities according to local or national guidelines.

### Outcomes

We included studies assessing any of the outcomes below [51, 52]. Since mortality is often the main outcome measure in MNCH studies, we selected it as the primary outcome [52]. We also included studies that had data from which we could calculate these outcomes.

1. Neonatal mortality is defined as deaths in the first 28 days of life among live births.
2. Perinatal mortality includes stillbirths and early neonatal deaths. Stillbirth is defined as the death of a fetus after 28 weeks of gestation but before delivery of the baby’s head. Early neonatal death is death of a neonate within seven days postpartum.
3. Maternal mortality is defined as the death of a woman during pregnancy or within 42 days of termination of pregnancy.

### Search Strategy

We searched articles in the following bibliographic databases: PubMed/Medline, POPLINE, EBSCO/CINAHL, Academic Search Complete, BiblioMap, ISI Web of Science, WHO regional database library, and the Cochrane Library. Additionally, we hand-searched relevant sources such as the Lancet series (Neonatal Survival and Maternal Survival) to enlarge the study pool. We also reviewed relevant internet sources from the WHO and Google Scholar for additional grey literature. Finally, we searched for additional studies using the snowball method of looking through the reference lists of retrieved articles. We limited the publication period to 15 years, from 1999 to 2014, to ensure that we retrieved a sufficient number of studies.

We used the following search strings combining the relevant medical subject headings (MeSH) with additional key/text words: (1) pregnant women, mothers, neonate, newborn, infant, or community; (2) maternal health or neonatal health; and (3) mortality or death. The specific electronic search strategy is provided in the S2 Text.

### Data Collection

#### Selection of studies

**Fig 1:**
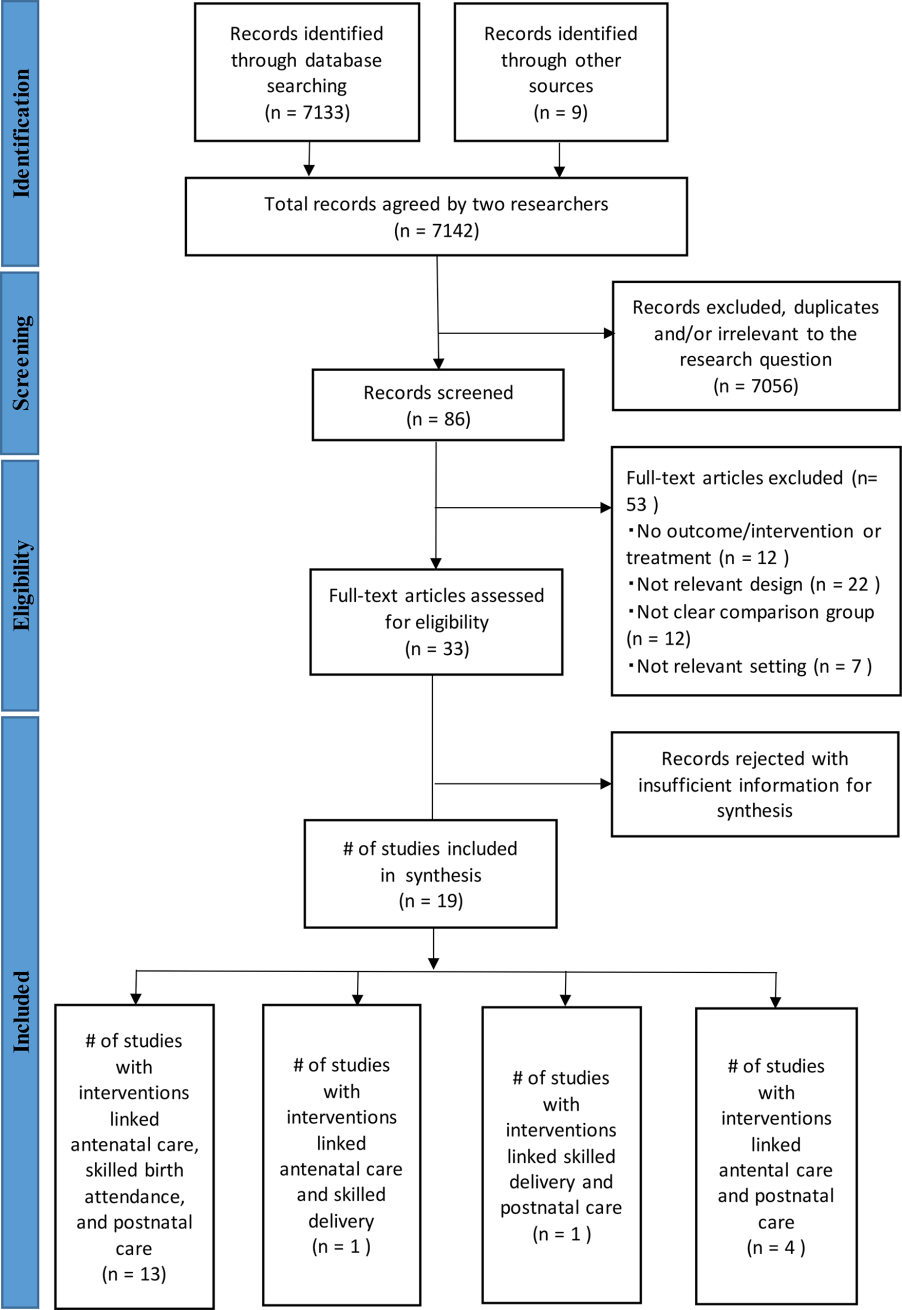
Diagram of information flow through phases of systematic review.

The study selection process is summarized in the flowchart in Fig 1. Two review authors (KK and SO) independently extracted data, and screened the quality and content of the included studies. We identified articles by analyzing the titles and abstracts for relevance and compliance with the selection criteria based on the research setting, study design, and reported outcomes. We classified articles as included, excluded, uncertain, or duplicate. We confirmed all potential included or uncertain studies and resolved any disagreements by consensus.

#### Data extraction and management

KK and SO extracted the features of each study (e.g., study design, setting, components of intervention package, and outcomes) and entered them into a standardized form. KK performed the extraction and SO checked for data accuracy and completeness. Again, when they noted discrepancies, the two review authors discussed until they reached an agreement, or consulted a third review author.

### Assessment of Risk of Bias on Included Studies

KK and SO assessed the quality of trials using the “risk of bias” tool presented in the Cochrane Handbook for Systematic Reviews of Interventions [45]. Specifically, we assessed the risk of bias in seven domains: sequence generation, allocation concealment, blinding of participants and outcome assessors, incomplete outcome data, selective outcome reporting, and other potential threats to validity. We resolved all discrepancies by consensus.

The summary of the risk of bias is shown in Table 1. Several studies lacked information related to the risk of bias or these risks were unclear. Due to the nature of the study design, allocation concealment was not an issue in the cluster-randomized studies we extracted [45]. However, we counted baseline imbalance in determining selection bias.

**Table 1:** Risk of bias for randomized and quasi-randomized studies.

	Azad 2010	Baqui (a) 2008	Baqui (b) 2008	Bhutta 2008	Bhutta 2011	Darmstadt 2010	Goudar2012	Jokhio 2008	Kirkwood 2013	Kumar 2008	Lewycka 2013	Mananhar 2004	Midhet 2010	More 2012	Persson 2013	Rahman 2011	Tripathy 2010	Colbourn 2013	Fottrell 2013
Random sequence generation (selection bias)	+	+	-	?	+	+	+	+	+	+	+	+	+	+	+	-	+	+	+
Allocation concealment (selection bias)	+	+	-	+	+	+	+	+	+	+	+	+	+	+	+	-	+	+	+
Blinding of participants and personnel (performance bias)	-	-	?	?	?	-	-	-	-	-	-	-	-	-	-	-	-	-	-
Blinding of outcome assessment (detection bias)	-	-	+	-	+	?	-	-	+	+	+	+	+	+	+	-	+	-	?
Incomplete outcome data (attrition bias)	+	+	?	?	?	+	+	+	+	+	+	+	-	+	-	+	+	-	-
Selective reporting (reporting bias)	+	+	-	+	+	+	+	+	+	+	+	+	-	+	+	-	+	+	+
Other bias	+	-	?	-	+	-	-	?	+	?	-	+	-	?	-	?	-	-	+

*Note*. +: low risk of bias;?: unclear risk of bias;-: high risk of bias.

### Measures of Treatment Effect

We present results as risk ratios (RR) with 95% confidence intervals (CI) for all randomized and quasi-randomized controlled trials.

### Unit of Analysis Errors

We checked whether the study contained a unit of analysis error. In cases where we observed this error, we re-analyzed the available data. No analysis error was found.

### Analysis

First, we narratively summarized the included study interventions. Second, we stratified the studies by the linkages of continuum of care time and space dimension stages. Then, we created a hierarchically categorized matrix by service delivery mode with the RRs of applied mortality. Third, we meta-analyzed neonatal, perinatal, and maternal mortality separately for each linkage of continuum of care time stages (antenatal care + skilled birth attendance + postnatal care; antenatal care + skilled birth attendance; skilled birth attendance + postnatal care; and antenatal care + postnatal care). Then, we analyzed all of these mortality types simultaneously to estimate a composite neonatal, perinatal, and maternal mortality risks for each linkage of time dimension stages. Finally, we stratified the studies by space dimension and estimated the composite risk of neonatal, perinatal, and maternal mortality.

The software RevMan 5.1 was used for the analysis. We assessed the heterogeneity of the meta-analyses from the P-values at a significance level of <0.10 and the I^2^ statistic. We used random-effects models to adjust for possible heterogeneity.

## Results

We retrieved a total of 7,142 articles from the sources (Fig 1). Of them, 3,046 studies were identified from the PubMed database, 4,087 through other databases, and nine through hand searching. After an initial screening, we excluded a total of 7,056 studies because they were duplicates or irrelevant to the research question. Of the 86 studies remaining, we excluded a further 53 because they had no outcome/intervention or treatment (n = 12), no relevant design (n = 22), no clear comparison group (n = 12), or no relevant setting (n = 7). We assessed the remaining 33 studies for eligibility through a review of the full-text articles. After this full-text review, we included a total of 19 studies in the meta-analysis [53–71]. They were all in English and had been published. Of the 19 studies, 17 were cluster randomized controlled trials (RCTs) [53–67, 70, 71], one was a pre-post intervention design [69], and one was a quasi-experimental study [68]. The included studies were mostly conducted in Asia and three were in Africa. In total, 361,306 births/live births were involved (Table 2).

**Table 2:** Summary of characteristics of studies.

Author/Country	Study design	Intervention to increase uptakes of MNCH care	Other interventions	Participants

		Description of intervention		
**Studies of linkage between antenatal care, skilled birth attendance, and postnatal care**				
Azad et al. 2010, Bangladesh	Cluster RCT	Participatory women's groups to improve maternal and neonatal health outcomes; TBAs training on safe deliveries and bag-valve-mask resuscitation of neonates with symptoms of birth asphyxia; basic and refresher clinical training relating to essential neonatal and maternal care for doctors, nurses, paramedical staff working at district.	N/A	I 15,695 births; C 15,257 births
Bhutta et al. 2011, Pakistan	Cluster RCT	Lady health workers' group sessions consisted of promotion of antenatal care and maternal health education, use of clean delivery kits, facility births, immediate newborn care, identification of danger signs. Lady health workers training for homevisit ANC within 24h of birth and on 3,7,14, 28 after delivery. TBA training for basic newborn care. Community health committees for maternal and newborn care.	N/A	I 12,517 births; C 11,568 births
Rahman et al. 2011, Bangladesh	Pre-post intervention with government service area	Clinical care impatient: (1) childbirth care (skilled obstetric care, essential newborn care, referral): (2) newborn baby and childcare (emergency care, case management for illness): (3) four focused ANC visits: (4) PNC. Family or community care: (5) pregnancy care (home-based life saving skills, pregnancy home visit at 12–14 wks and 32–34 wks): (6) PNC home visit at 0,3,7 and 28 days.	N/A	I 5,305 births; C 4,816 births
Baqui et al. 2008 (a), India	Quasi experimental study	Homevisit by auxiliary nurse-midwife including promotion of at least 3 ANC for mothers/families; encourage families to call auxiliary nurse-midwife or trained TBAs to attend delivery; home visit PNC as soon as possible after birth during 0–27 days; follow-up visits for sick.	N/A	I 7,812 livebirths; C 6,014 livebirths
Baqui et al. 2008 (b), Bangladesh	Cluster RCT	Community health workers training; Home-care model service delivery group: community health workers provide ANC homevisit, PNC visit, referral of sick newborns to health facility and treatment in the house with injectable antibiotics: Community-care model service delivery group: TBA orientation on cleanliness during delivery, danger sign, newborn care, community meeting with pregnant women by community mobilisers.	(1) Home-care model service delivery group; (2) Community-care model service delivery group; (3) Control group	Homecare 15,370 births; Community-care 16,908 births; C 15,914 births
Jokhio et al. 2005, Pakistan	Cluster RCT	Obstetricians and female paramedics trained TBAs (advice on ANC, intrapartum care, PNC; clean delivery; use of disposable delivery kits; refer women for emergency obstetrical care; care of the newborn); ask women to visit at least three times during pregnancy; Linkage of TBA with lady health workers. Outreach clinics for ANC.	N/A	I 10,093 births; C 9,432 births
Persson et al. 2013, Vietnam	Cluster RCT	Laywomen facilitated monthly meeting in groups composed of CHC staff (accountable for the health care in the community) and key persons (vice chairman and the Women Union leader) in the communes. The key persons act as facilitators in supporting CHC staff and make efforts to improve health care practice. A problem-solving approach was employed.	N/A	I 11,906 births; C 10,655 births
Kumar et al. 2008, India	Cluster RCT	Community health workers provided an essential newborn care package (birth preparedness, clean delivery and cord care, thermal care, breastfeeding promotion, danger sign recognition); provided a liquid crystal hypothermia indicator (ThermoSpot) by community health workers through collecitve meeting; community meetings, folk song meetings, community volunteer meetings; two ANC and two PNC home visits.	(1) Group received an essential newborn care package; (2) Group received the essential newborn care package plus use of a ThermoSpot; (3) Control group	Essential newborn care 1,581 births; Essential newborn care +ThermoSpot 1,135 births; C 1,143 births
Tripathy et al. 2010, India	Cluster RCT	Training of facilitators who activate and strengthen groups, support them in identifying problems, help to plan possible solutions and support the implementation and monitoring of solution strategies in the community.	N/A	I 9,686 births; C 9,089 births
Manandhar et al. 2004, Nepal	Cluster RCT	Female facilitators convened 9 women’s group meetings every month. The facilitator supported groups through an action-learning cycle to activate and strengthen existing mothers' groups, support them in identifying and prioritising maternal and neonatal problems, help to identify possible solutions and support the planning, implementation and monitoring of the solution strategies in the community; resuscitaires, phototherapy units, warm cots to primary health centres; training in essential newborn care for government health staff and female community health volunteers and TBAs.	N/A	I 2,972 births; C 3,303 births
More et al. 2012, India	Cluster RCT	Recruit full-time facilitators (*sakhi*)and provide training on a range of health care topics; the facilitators conducted 36-meeting cycle emphasising on knowing what services were available, choosing appropriate perinatal health care, understanding best practice, and negotiating optimal care with family and providers	N/A	I 9,155 births; C 9,042 births
**Studies of linkage between antenatal care and skilled birth attendance**				
Midhet and Becker, 2010, Pakistan	Cluster RCT	Female volunteers training to educate women using IEEC materials (cover preparation for pregnancy and delivery, nutrition, danger signs during pregnancy, delivery and postpartum) for increasing awareness of safe motherhood and neonatal health. Educate husbands using IEEC for husband. TBA training for clean delivery and recognition of complications; set up of emergency transportation system to local health facility or district hospital using public transport vehicles or VHF wireless telecommunication system; Traning for obstetricians, pediatricians and anaesthetists working at the district hospital.	(1) Women's IEEC only group; (2) Couples' IEEC group; (3) Control	Women's IEEC 836 births; Couples' IEEC 703 births; C 1,022 births
**Studies of linkage between skilled birth attendance and postnatal care**				
Gouder et al. 2012, India	Cluster RCT	WHO essential newborn care training for physicians, nurse, midwives, TBAs (initial care for neonates following birth and during the first week after birth, initiation of breathing and resuscitation, thermoregulation, early and exclusive breastfeeding, skin-to-skin care, small baby care, baby care and danger signs counseling, initial management of complication).	N/A	I 6,409 births; C 6.386 births
**Studies of linkage between antenatal care and postnatal care**				
Lewycka et al. 2013, Malawi	Cluster RCT	Training for women's group to identifiy and prioritise MCH problem, identify strategies to implement, plan and implement them, and assess them and make plan for the future; training volunteer peer counsellers made home visits 5 times (third trimester, 1 week after birth, 1, 3, 5 months) for health education (EBF, infant care, immunisations, PMTCT, FP); supervision of counsellors by health surveillance assistants of district health office.	(1) Women's group + volunteer peer counselling; (2) Women's group only; (3) Volunteer peer counseling only; (4) Control	Women ' group + volunteers 4,601 births; Women's group 4,773 births; Volunteers 4,690 births; C 5,059 births
Darmstadt et al. 2010, Bangladesh	Cluster RCT	Community health workers promoted birth and newborn care preparedness through two ANC home visits at 12–16 and 32–34 weeks of gestation; PNC home visits on days 0, 2, 5, 8; refer sick neonates to hospital.	N/A	I 4,729 births; C 5,350 births
Bhutta et al. 2008, Pakistan	Cluster RCT	ANC Promotion, LHW visit pregnant women 2 times during pregnancy, within 24h postpartum, 4 times in the first postnatal month and link up with local Dais. All health-care facilities were provided with basic and intermediate newborn care equipment by UNICEF, special training for medical and nursing staff	N/A	I 3,064 births; C 2,778 births
Kirkwood et al. 2013, Ghana	Cluster RCT	Training CBSVs; identify pregnant women in the community; two home visits during pregnancy; three PNC home visits on days 1, 3 and 7 (incl. referral of sick baby); developing a sustainable supervisory and remuneration structure for the CBSVs; sensitisation of health facility staff to intervention messages and approach; sensitisation of TBAs.	N/A	I 22,732 births; C 22,436 births

*Note*. ANC = antenatal care; C = control arm; CBSV = community-based surveillance volunteer; CHC = community health care; FP = family planning; I = intervention arm; IEEC = information and education for empowerment and change; LHW = lady health worker; MCH = maternal and child health; N/A = not applicable; PMTCT = prevention for mother-to-child transmission; PNC = postnatal care; RCT = randomized controlled trial; TBA = traditional birth attendant.

**Fig 2:**
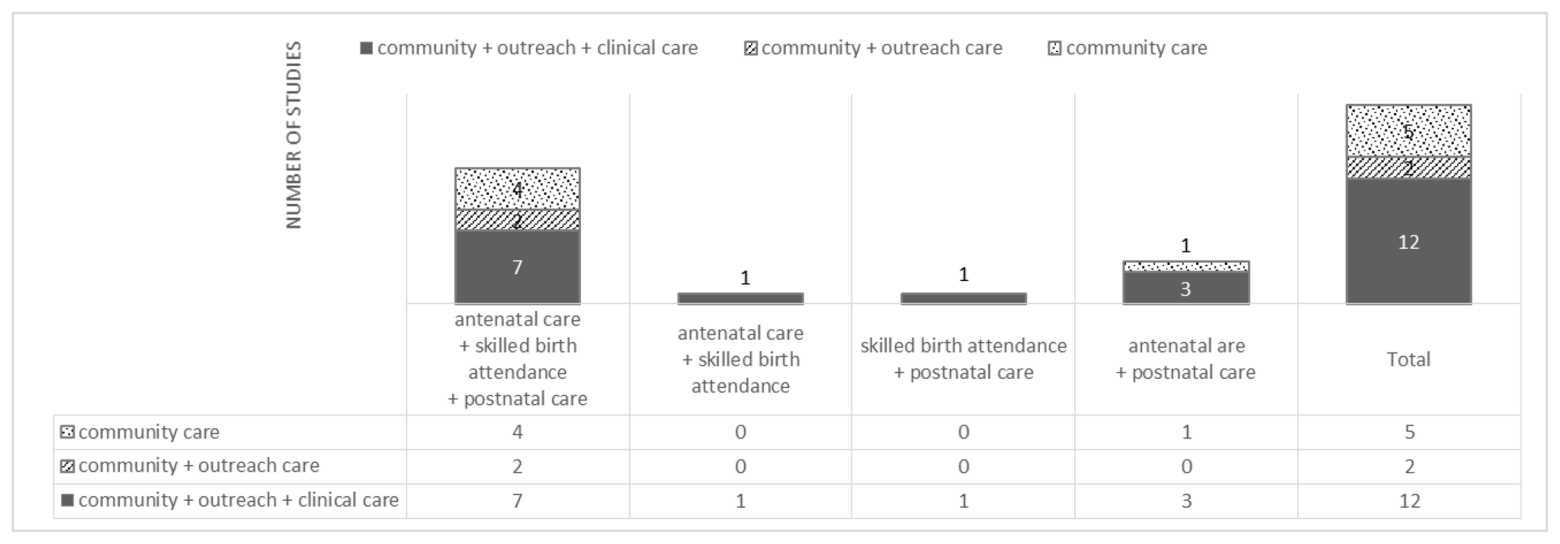
Number of studies in each category of continuum of care linkage.

As shown in Fig 2, out of the 19 studies, 13 involved interventions that linked antenatal care, skilled birth attendance, and postnatal care [53, 54, 56, 59, 61, 63, 65–69]. One study each linked antenatal care and skilled birth attendance [64] or skilled birth attendance and postnatal care [58]. Four studies linked antenatal care and postnatal care [55, 57, 60, 62]. However, no study used interventions that linked all the time stages as recommended—namely, four antenatal care visits; skilled birth attendance; and postnatal care within 48 hours, at seven days, and at six weeks.

Regarding the space dimension, among the 19 studies, 12 used interventions that linked community–family care, outpatient–outreach care, and clinical care [53–60, 62, 64, 68, 69]; two studies included interventions that linked community–family care and outpatient–outreach care [61, 66], and five studies included interventions on community–family care only [62, 63, 65, 67].

### Timing and Frequency of Antenatal Care/Postnatal Care

The timing and frequency of antenatal care or postnatal care services delivery differed among the included studies. Most of the studies involved four antenatal care visits, with the minimum numbers of antenatal care visits for the interventions ranging from one to four times during the pregnancy period. The timings of antenatal care, however, were not defined in most of the studies—indeed, only three studies reported the timings of antenatal care: twice at 12–16 weeks and 32–34 weeks in a study in Bangladesh [54], twice during the first and second trimesters in a study in Pakistan [64], and once in the third trimester in a study in Malawi [62].

In the case of postnatal care, the timings varied, ranging from zero days to five months. However, in most studies, postnatal care was provided for seven days at the very least. The frequency was also rather diverse, ranging from one to five times. Six studies did not make clear mention of the timing or frequency, but reported on interventions during the postnatal care period.

### Effectiveness of Care Linkages between Antenatal Care, Skilled Birth Attendance, and Postnatal Care

Table 3 shows the results of the 13 studies that linked antenatal care, skilled birth attendance, and postnatal care. Except for one study conducted in Malawi, all studies were conducted in Asia (India, Bangladesh, Vietnam, Nepal, and Pakistan). Of the 13 studies, six included interventions that linked the three stages of the space dimension (i.e., community–family care, outpatient–outreach care, and clinical care). Although they linked the three time stages, the frequency of antenatal care visits ranged from two to four times between the studies. The frequencies and timings of postnatal care were not the same across studies.

**Table 3:** Studies of linkage between antenatal care, skilled birth attendance, and postnatal care.

Author/Country	Space dimension			Time dimension			Cares of interest			Mortality RR [95% CI]		
	Community-Family care	Outpatient-outreach care	Clinical care	ANC	Birth	PNC	ANC	Birth	PNC	Neonatal mortality	Perinatal mortality	Maternal mortality
Azad et al. 2010, Bangladesh	+	+	+	+	+	+	≥4 times	Yes (by health workers or TBAs)	Yes, but no clear frequency and timing	0.90 [0.80–1.01]	0.97 [0.89–1.06]	1.67 [1.08–2.58]
Bhutta et al. 2011, Pakistan	+	+	+	+	+	+	2 times	Yes	5 times (24h, 3 days, 7 days, 14 days, 28 days postpartum)	0.88 [0.78–0.99]	0.84 [0.78–0.91]	
Rahman et al. 2011, Bangladesh	+	+	+	+	+	+	4 times	Yes	3 times (0 day, 3 days, 7 days postpartum)		0.57 [0.47–0.68]	
Baqui et al. 2008, (a) India	+	+	+	+	+	+	≥3 times	Yes (by auxiliary nurse-midwife or TBAs)	2 times (2 days between 0–27 days postpartum)	0.84 [0.75–0.93]		
Baqui et al. 2008, (b) Bangladesh	+	+	+	+	+	+	2 times (12–16 wks and 32–34 wks)	Yes (by referral or treatment in the house if necessary)	3 times (1 day, 3 days, 7days postpartum)	1.01 [0.87–1.17]		
Jokhio et al. 2005, Pakistan	+	+	+	+	+	+	3 times	Yes (by TBA)	Yes, but no clear frequency and timing	0.72 [0.62–0.82]	0.71 [0.66–0.78]	0.74 [0.45–1.23]
Persson et al. 2013, Vietnam	+	+	-	+	+	+	Yes, but no clear frequency and timing	Yes	Yes, but no clear frequency and timing	0.90 [0.74–1.09]	0.89 [0.76–1.07]	0.22 [0.02–2.00]
Kumar et al. 2008, India	+	+	-	+	+	+	2 times	Yes	2 times (24h, 3 days postpartum)	0.51 [0.39–0.67]	0.63 [0.51–0.78]	
Tripathy et al. 2010, India	+	-	-	+	+	+	≥3 times	Yes	1 time (7 days postpartum)	0.72 [0.63–0.82]	0.8 [0.73–0.90]	0.77 [0.53–1.12]
Manandhar et al. 2004, Nepal	+	-	-	+	+	+	Yes, but no clear frequency and timing	Yes	Yes, but no clear frequency and timing	0.71 [0.54–0.94]	0.93 [0.74–1.18]	0.20 [0.04–0.91]
More et al. 2012, India	+	-	-	+	+	+	3 times	Yes	Yes, but no clear frequency and timing	1.48 [0.74–0.94]		

*Note*. + = yes;— = no; RR = risk ratio; ANC = antenatal care; PNC = postnatal care; RR = relative risk; TBA = traditional birth attendant.

As for the components of the intervention, we noted some particular characteristics. For instance, primary-care-level health officers (e.g. lady health workers, community health workers) provided antenatal care or postnatal care within the community for women and children via home visits [54, 56, 61, 68, 69] and outreach services [59]. To improve care delivery, traditional birth attendants (TBAs) received training in the form of basic neonatal care, cleanliness during delivery, and referral of women for emergency obstetric care [54, 56, 59, 68]. Interventions often included community meetings for women to promote MNCH. The facilitators of such interventions were TBAs, community health workers, or other recruited facilitators [54, 56, 61, 65, 67].

The intervention packages in these 13 studies resulted in significant reductions in mortality. Twelve studies assessed neonatal deaths. Among them, seven reported significant reductions in neonatal mortality as a result of the interventions. Furthermore, 10 of the 13 studies measured perinatal deaths. Of them, five reported significant reductions in perinatal mortality as a result of the interventions. Six studies measured maternal deaths. Of them, one study showed a significant reduction as a result of the intervention and one study showed a significant increase.

**Fig 3:**
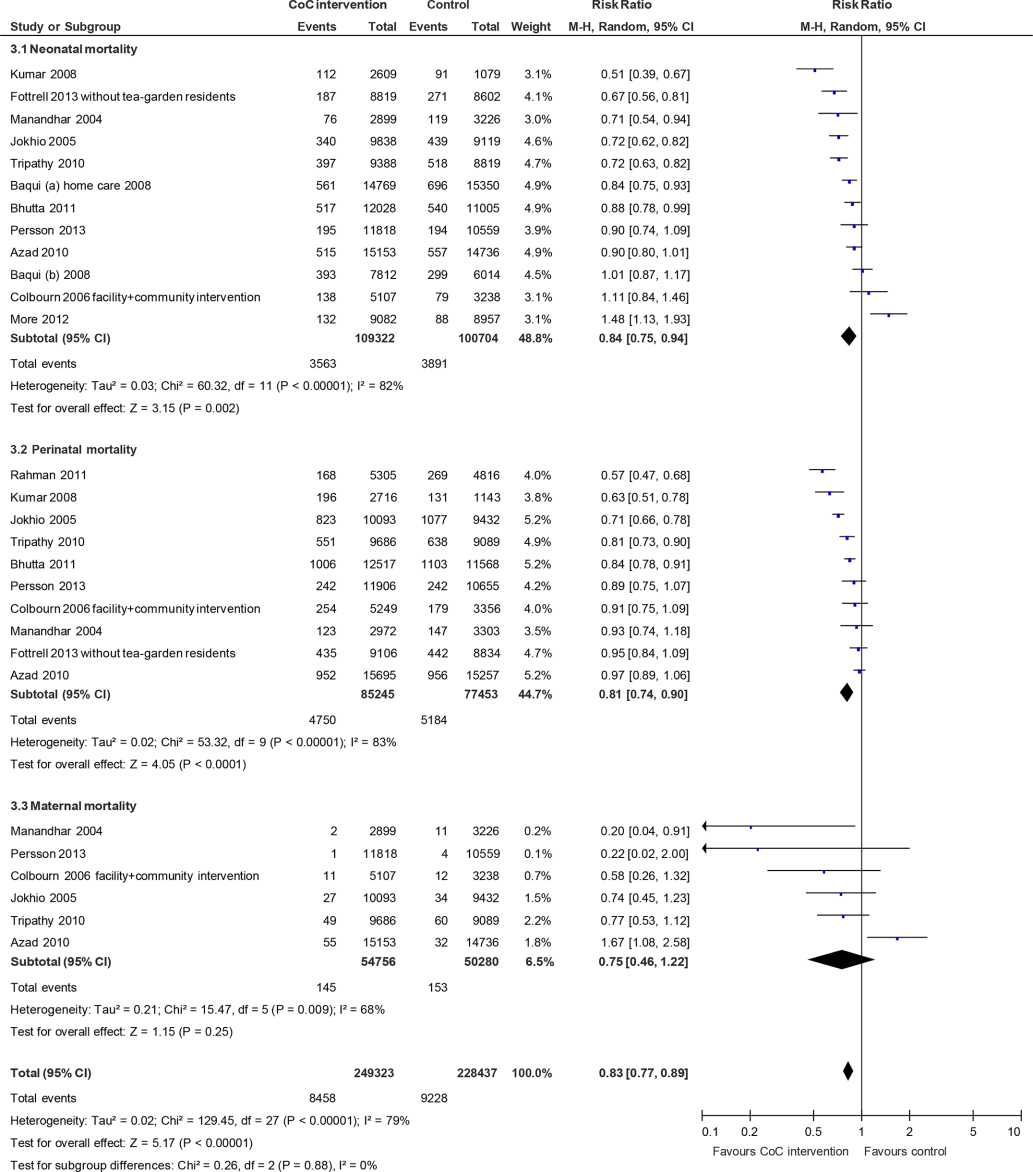
Mortality risk ratio for interventions linking antenatal care, skilled birth attendance, and postnatal care.

**Fig 4:**
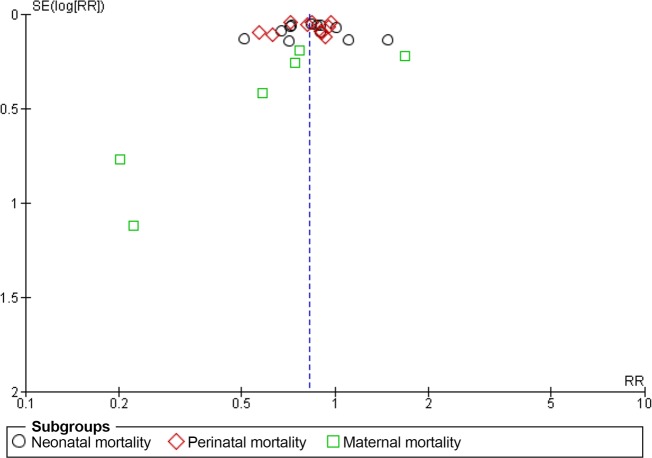
Funnel plot of studies linking antenatal care, skilled birth attendance, and postnatal care.

According to the meta-analysis, the interventions that linked antenatal care, skilled birth attendance, and postnatal care significantly reduced neonatal mortality (RR 0.84; 95% CI 0.75 to 0.94, random effects [12 studies, n = 184,260], I^2^ 82%, P < 0.01) and perinatal mortality (RR 0.81; 95% CI 0.74 to 0.90, random effects [10 studies, n = 136,153], I^2^ 83%, P < 0.01) (Fig 3). However, the interventions did not lead to a significant decrease in maternal mortality (RR 0.75; 95% CI 0.46 to 1.22, random effects [six studies, n = 96,691], I^2^ 68%, P < 0.01). The composite measure of all three mortality types was significantly reduced (RR 0.83; 95% CI 0.77 to 0.89, random effects, I^2^ 79%, P < 0.01). To explore the considerable heterogeneities observed in this meta-analysis, we explored the publication bias using a funnel plot (Fig 4). The included studies were symmetrically distributed on both sides of the combined effect size line, indicating no obvious publication bias.

### Effectiveness of Care Linkage between Antenatal Care and Skilled Birth Attendance

Table 4 shows the result of the one reviewed study that linked antenatal care and skilled birth attendance [64]. This study was a cluster RCT conducted in Pakistan. The study also linked the space dimension stages (i.e., community–family care, outpatient–outreach care, and clinical care). The intervention included sensitization for mothers only or for couples, TBA training for clean delivery and complication recognition, and emergency referral through vehicles or very high frequency communication systems.

**Table 4:** Studies of linkage between antenatal care and skilled birth attendance.

Author	Space dimension			Time dimension			Cares of interest			Mortality RR [95% CI]		
	Community-Family care	Outpatient-outreach care	Clinical care	ANC	Birth	PNC	ANC	Birth	PNC	Neonatal mortality	Perinatal mortality	Maternal mortality
Midhet and Becker, 2010, Pakistan	+	+	+	+	+	-	2 times (1st/2nd trimesters)	Yes	No	0.68 [0.41–1.10]	0.51 [0.38–0.68]	

*Note*. + = Yes;— = No; ANC = antenatal care; PNC = postnatal care; RR = risk ratio.

The intervention in this study did not lead to any significant reductions in neonatal mortality (RR 0.68; 95% CI 0.41 to 1.10); however, we identified significant reductions in perinatal mortality (RR 0.51; 95% CI 0.38 to 0.68) (Table 4). No data were available for maternal mortality. We did not perform a meta-analysis for this intervention because of the lack of other studies providing impact estimates on mortality.

### Effectiveness of Care Linkage between Skilled Birth Attendance and Postnatal Care

Table 5 shows the result of the one reviewed study that linked skilled birth attendance and postnatal care [58]. The study was a cluster RCT conducted in India. The study included the three space dimension stages, and included interventions involving newborn care training for physicians, nurses, midwives, and TBAs.

**Table 5:** Studies of linkage between skilled birth attendance and postnatal care.

Author	Space dimension			Time dimension			Cares of interest			Mortality RR [95% CI]		
	Community-Family care	Outpatient-outreach care	Clinical care	ANC	Birth	PNC	ANC	Birth	PNC	Neonatal mortality	Perinatal mortality	Maternal mortality
Gouder et al. 2012, India	+	+	+	-	+	+	No	Yes	Yes, but no clear frequency and timing		0.91 [0.75–1.11]	

*Note*. + = Yes;— = No; ANC = antenatal care; PNC = postnatal care; RR = risk ratio.

This study measured only perinatal mortality, which the intervention did not significantly reduce (RR 0.91; 95% CI 0.75 to 1.11) (Table 5). We did not perform a meta-analysis because of the lack of studies providing impact estimates on mortality.

### Effectiveness of Care Linkage between Antenatal Care and Postnatal Care

Table 6 shows the result of the four studies that linked antenatal care and postnatal care, but not skilled birth attendance. Two studies were conducted in Asia (Bangladesh and Pakistan) and the other two in Africa (Malawi and Ghana). All studies were cluster RCTs. Of them, three studies had interventions that linked all three continuum of care space stages, while the remaining study was concerned only with community–family care. The frequency of antenatal care visits ranged from one to three times among the studies. The frequencies and timings of postnatal care also differed across the studies.

**Table 6:** Studies of linkage between antenatal care and postnatal care.

Author	Space dimension			Time dimension			Cares of interest			Mortality RR [95% CI]		
	Community-Family care	Outpatient-outreach care	Clinical care	ANC	Birth	PNC	ANC	Birth	PNC	Neonatal mortality	Perinatal mortality	Maternal mortality
Bhutta et al. 2008, Pakistan	+	+	+	+	-	+	2 times	No	4 times (24h, 7 days, 14 days, 28 days postpartum)	0.69 [0.55–0.87]	0.72 [0.61–0.85]	
Darmstadt et al. 2010, Bangladesh	+	+	+	+	-	+	2 times (12–16 wks and 32–34 wks)	No	4 times (0 day, 2 days, 5 dsays, 8 days postpartum)	0.86 [0.68–1.10]		
Kirkwood et al. 2013, Ghana	+	+	+	+	-	+	2 times	No	3 times (1 day, 3 days, 7 days postpartum)	0.99 [0.89–1.09]		
Lewycka et al. 2013, Malawi	+	-	-	+	-	+	1 time (3rd trimester)	No	4 times (7 days, 1 month, 3 month, 5 month postpartum)	0.70 [0.54–0.90]	0.77 [0.63–0.93]	0.87 [0.51–1.51]

*Note*. + = Yes;— = No; ANC = antenatal care; PNC = postnatal care; RR = risk ratio.

As for the components of the interventions, all four studies included home visits for antenatal care or postnatal care. The practitioners who performed the home visits were primary-level health workers [55, 57], community volunteers [60], or trained women’s group members [62].

This intervention linkage resulted in significant reductions in mortality. All four studies measured neonatal deaths, but only two reported a reduction in neonatal mortality. Two of the four studies measured perinatal mortality, both reporting significant reductions as a result of the intervention. Maternal death was measured in one of the four studies, but was not significantly reduced [62].

**Fig 5:**
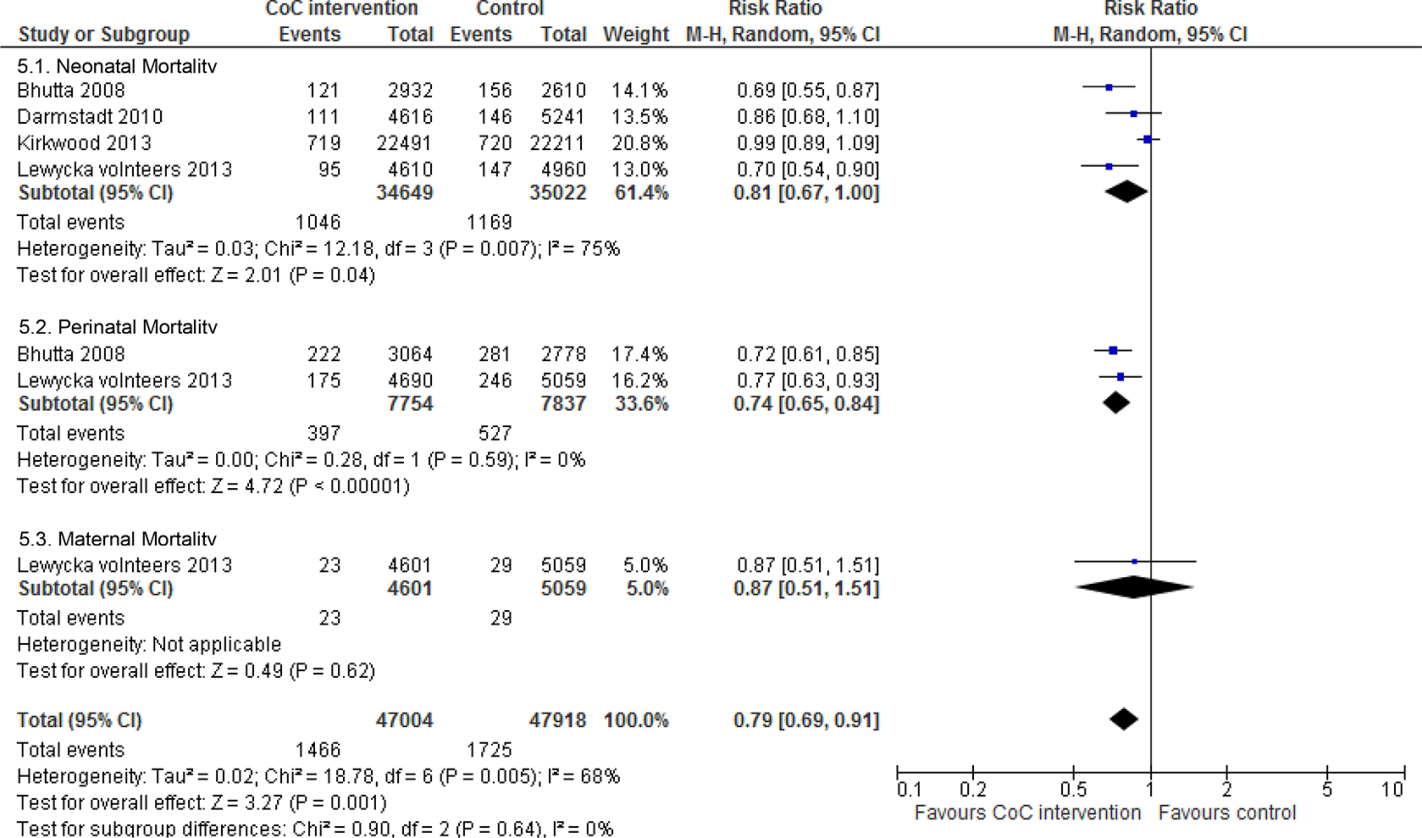
Mortality risk ratio for interventions linking antenatal care and postnatal care.

Fig 5 shows the meta-analysis. Neonatal mortality (RR 0.81; 95% CI 0.67 to 1.00, random effects [four studies, n = 69,671], I^2^ 75%, P < 0.01) and perinatal mortality (RR 0.74; 95% CI 0.65 to 0.84, random effects [two studies, n = 15,591], I^2^ 0%, P < 0.01) were both significantly decreased in this linkage. We did not perform a meta-analysis for maternal mortality due to a lack of studies. The interventions employing a linkage of antenatal care and postnatal care significantly reduced a composite of the three mortality types (RR 0.79; 95% CI 0.69 to 0.91, random effects, I^2^ 68%, P = 0.005).

### Effectiveness of Care Linkage between Time and Space Dimensions

**Fig 6:**
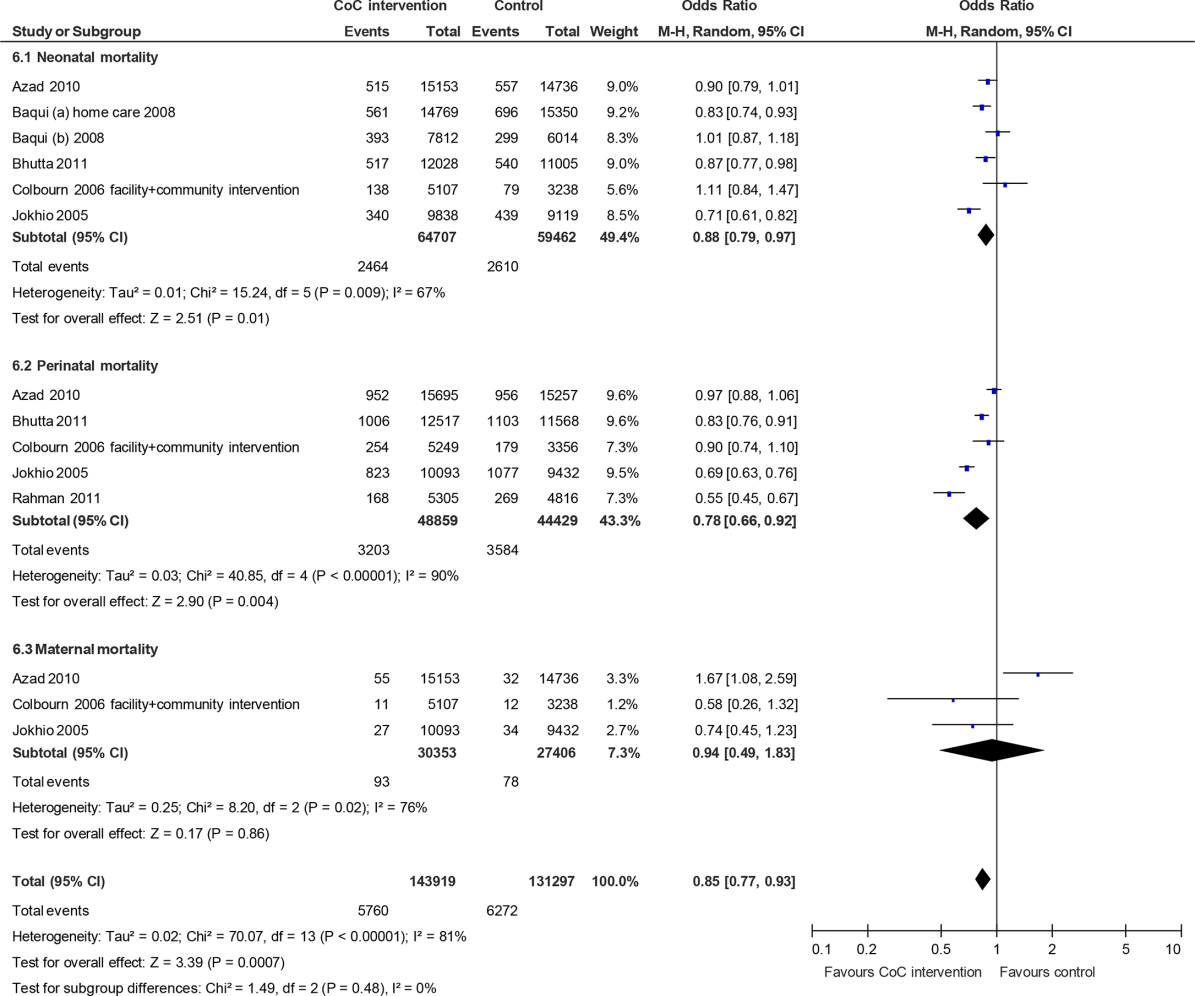
Mortality risk ratio for interventions linking the three stages of the time dimension (antenatal care, skilled birth attendance, and postnatal care) and the three stages of the space dimension (community-family care, outpatient-outreach care, and clinical care).

**Fig 7:**
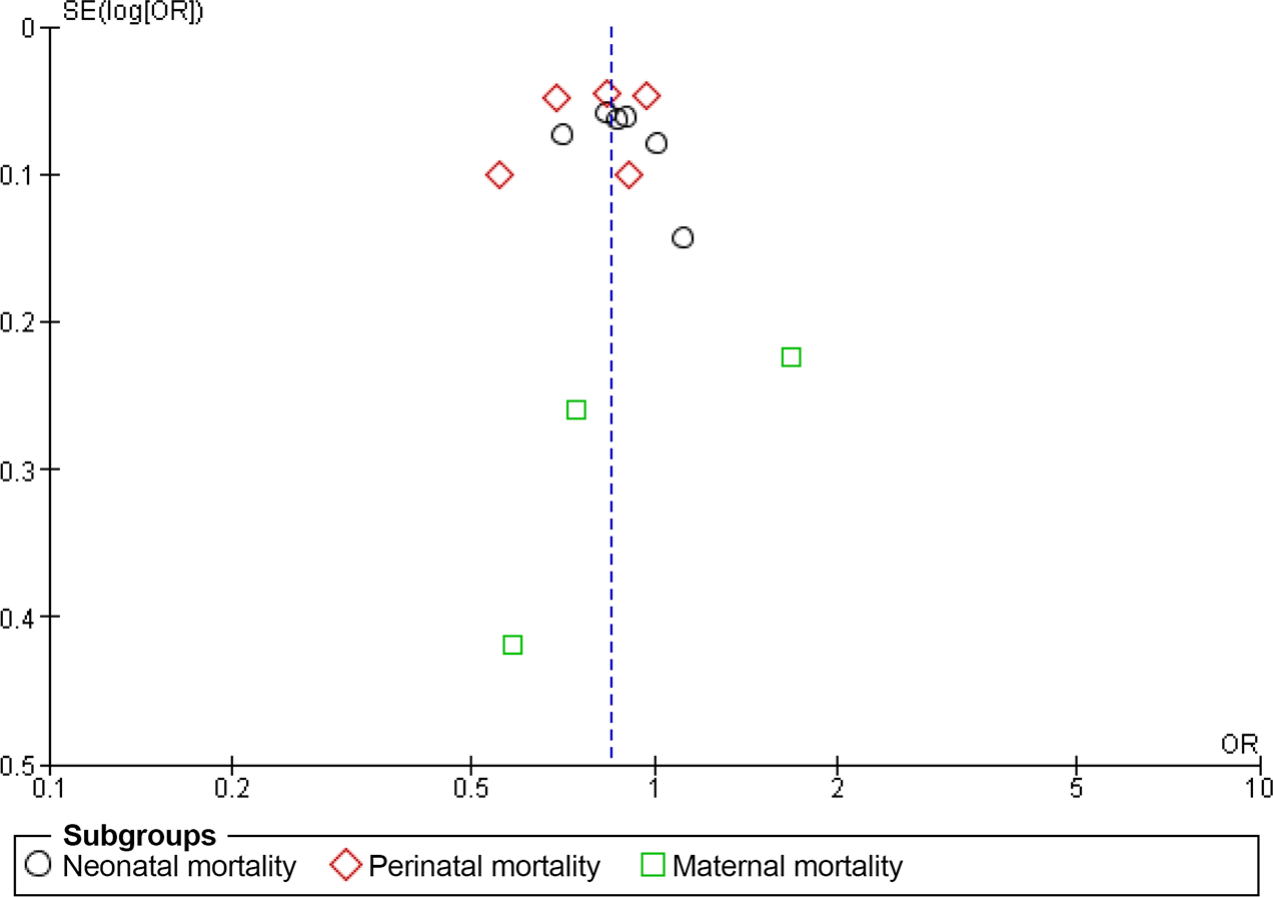
Funnel plot of studies linking the three stages of the time dimension (antenatal care, skilled birth attendance, and postnatal care) and the three stages of the space dimension (community-family care, outpatient-outreach care, and clinical care).

Six randomized studies linked the three stages of the time dimension (antenatal care, skilled birth attendance, and postnatal care) and the three stages of the space dimension (community–family care, outpatient–outreach care, and clinical care). According to the meta-analysis results including those studies, we observed significant reductions for neonatal mortality (RR 0.88; 95% CI 0.79 to 0.97, random effects [six studies] I^2^ 67%, P < 0.01) and perinatal mortality (RR 0.78; 95% CI 0.66 to 0.92, random effects [five studies] I^2^ 90%, P < 0.01). However, we did not observe significant reductions in maternal mortality (RR 0.94; 95% CI 0.49 to 1.83, random effects [three studies] I^2^ 76%, P = 0.02). A composite measure of mortality was also significantly reduced (RR 0.85; 95% CI 0.77 to 0.93, random-effects, I^2^ 81%, P < 0.01) (Fig 6). We did not find any obvious asymmetry in the funnel plot (Fig 7).

## Discussion

This is the first systematic review to examine the effectiveness of interventions combining the continuum of care time and space dimensions for maternal, neonatal, and perinatal mortality. We included a total of 19 studies in this review; several results of some importance were obtained.

First, none of the studies followed the standards of the continuum of care time dimension stages: four antenatal care visits; use of skilled birth attendance; and postnatal care within 48 hours, at seven days, and at six weeks [31]. However, two-thirds of the included studies did contain interventions that linked antenatal care skilled birth attendance, and postnatal care, albeit without considering frequency and timing of care; another two-thirds of the studies linked community–family care, outpatient–outreach care, and clinical care. Second, the interventions linking antenatal care, skilled birth attendance, and postnatal care led to significant decreases in the composite measure of neonatal, perinatal, and maternal mortality. However, the maternal mortality results were inconsistent. The composite measure of mortality also showed reductions in a meta-analysis of the interventions involving linkages between the time and space dimension stages.

### Linkage Patterns of Time and Space Dimensions

As mentioned above, none of the studies followed the standards of continuum of care. However, without taking frequency and timing into consideration, linking the three time dimension stages reduced the risk of neonatal and perinatal deaths. In a recent systematic review, the perinatal mortality increased with reduced antenatal care visits, but the difference was of borderline statistical significance (three studies: RR 1.15; 95% CI 1.01 to 1.32) [24]. This could be because taking antenatal care would positively affect skilled birth attendance [72]. This may also support strategies that promote “goal-oriented” antenatal care visits [24, 49], which directs attention to the quality of antenatal care, including its components and timing, more than the quantity of such care.

In our review, the frequencies and timing of postnatal care differed across studies. This may be because of the postnatal care changes in global recommendations by the WHO [30, 41, 42]. In our study, most of the postnatal care interventions included the first postnatal care visit within 1–2 days. However, the timing of additional postnatal care visits was not consistent. The risk of death is significantly elevated even after 42 days postpartum [73, 74]. Regarding the frequency of postnatal care, there was no compelling evidence that more postnatal care visits are associated with fewer neonatal or maternal deaths [75, 76]. Decisions on the frequency and timing of postnatal care could be better made if they were defined according to local needs [76].

All of the included studies contained community–family care in the space dimension, and two-thirds included links with outpatient–outreach care and clinical care. Community involvement is a positive factor, especially for reducing neonatal mortality, perinatal mortality, maternal morbidity, and the number of stillbirths, as demonstrated in a previous review [77].

### Effective Linkages of the Time Dimension for Reducing Mortality

#### Linkage between antenatal care, skilled birth attendance, and postnatal care

According to our meta-analysis, interventions that linked all three stages of the time dimension were most effective. Interventions utilizing a linkage of antenatal care, skilled birth attendance, and postnatal care appeared to reduce the risk of neonatal and perinatal deaths. Only the risk of maternal deaths showed inconsistent results. The study by Azad, which showed an increase in maternal deaths due to the intervention, did not provide a specific reason for this result. However, no deaths among mothers who attended women’s groups, making it difficult to consider that the intervention would have influenced the negative results in maternal mortality. Notably, the significant increase was observed only when marginal populations were included; thus, omission error could have affected the intervention results. However, the intervention linkage of antenatal care, skilled birth attendance, and postnatal care was found to be effective for a composite measure of neonatal, perinatal, and maternal mortality. This suggests the importance of considering these three time stages in designing MNCH interventions in LMICs.

#### Linkage between antenatal care and postnatal care

The intervention packages that combined only antenatal care and postnatal care significantly reduced the risk of neonatal and perinatal mortality. This linkage was also effective in reducing the composite measure of mortality. Antenatal care is an entry point to MNCH care, suggesting that antenatal care may improve overall MNCH care uptake, which leads to better MNCH outcomes. However, this result also suggests that postnatal care might complement the effectiveness of antenatal care. Thus, linkages of antenatal care with postnatal care could be a key to designing effective MNCH interventions.

### Effective Linkages of the Time and Space Dimensions for Reducing Mortality Risks

In the meta-analysis, the linkage of all time and space stages was effective for reducing neonatal, perinatal, and maternal mortality. This was among the primary evidence found in this review study. The importance of both dimensions has been argued in the past, however, until now, evidence justifying the effectiveness of linking the continuum of care time and space dimensions has been scarce. Indeed, the continuum of care has been promoted without solid evidence of its effectiveness. Understandably, further trial evidence will be necessary to demonstrate the effectiveness of the continuum of care with high reliability. Nevertheless, our systematic review will serve as evidence in defending the effectiveness of the continuum of care.

### Suggested Definition and Intervention of Continuum of Care

The findings of this review demonstrate the effective continuum of care linkages: thus we can use the results to better define the components of continuum of care and thereby exploit it for the design of future intervention. For the time dimension, the basic components of continuum of care could be linkages between antenatal care, skilled birth attendance, and postnatal care. However, interventions combining antenatal care with postnatal care would be also an effective option when resources are limited. The frequency of antenatal care should range between two and four visits. We could not clearly identify the timings of antenatal care in this systematic review. For postnatal care, timing appears more important than does frequency; since most of the included studies first implemented postnatal care at 0–2 days postpartum, this postnatal care timing should be at least considered. As for delivery, ensuring the presence of skilled birth attendants is not always possible. However, if TBAs receive training in appropriate skills, said TBAs could be included as assistants for post-delivery care.

For the space dimension of the continuum of care, a basic component of interventions is linkages between community–family care, outpatient–outreach care, and clinical care. We particularly recommend interventions based on community involvement, as per previous evidence.

Additionally, our review and meta-analysis results indicated that interventions that include home visit care would be effective for improving neonatal and perinatal health. Particularly, early postnatal care visits between 0 and 2 days would be important. These results suggest that a community-focused intervention would be a favorable design.

### Limitations

This systematic review has two limitations. First, the lack of randomized studies may have obscured the real results of the meta-analysis. In particular, we found only one study each including interventions that linked antenatal care and skilled birth attendance or skilled birth attendance and postnatal care. Thus, for this linkage, we could not conduct a meta-analysis. Further evidence of these linkages is required to confirm their effectiveness.

Second, a number of groups showed significant statistical heterogeneity. We could have excluded studies that conflicted with the other studies from the meta-analysis. However, we could not identify the sources of heterogeneity; indeed, even after excluding the outlying studies, we identified heterogeneity. Furthermore, eliminating studies would also be unwise because it can introduce bias [45]. Thus, the findings need to be interpreted with caution.

## Conclusions

Our review encourages continuous uptake of antenatal care, skilled birth attendance, and postnatal care to improve MNCH outcomes in LMICs. The review was conclusive regarding the reduction of neonatal and perinatal deaths. Although maternal deaths were not significantly reduced, composite measures of all mortality were. As such, the evidence is sufficient to scale up this intervention package for the improvement of MNCH outcomes.

## Supporting Information

S1 Table PRISMA 2009 Checklist. (DOC)

S1 Text Research protocol. (DOCX)

S2 Text Search Terms for “Effective Linkages of Continuum of Care at Improving Neonatal, Perinatal, and Maternal Mortality” Review–Published Literature (DOCX)
