# Supplementary material for: Effective Linkages of Continuum of Care for Improving Neonatal, Perinatal, and Maternal Mortality: A Systematic Review and Meta-Analysis
Source: PLoS One. 2015 Sep 30;10(9):e0139288. doi: 10.1371/journal.pone.0139288 (PMC4589290; doi:10.1371/journal.pone.0139288)
Supplement: S1 Text — (DOCX) [file pone.0139288.s002.docx]

**PROSPERO International prospective register of systematic reviews**

# Review title and timescale

## Review title

Give the working title of the review. This must be in English. Ideally it should state succinctly the interventions or exposures being reviewed and the associated health or social problem being addressed in the review.

Effectiveness of the continuum of care on maternal and newborn mortality, morbidity, and health behavior: a systematic review

## Original language title

For reviews in languages other than English, this field should be used to enter the title in the language of the review. This will be displayed together with the English language title.

## Anticipated or actual start date

Give the date when the systematic review commenced, or is expected to commence. 01/11/2013

## Anticipated completion date

Give the date by which the review is expected to be completed. 31/12/2013

## Stage of review at time of this submission

Indicate the stage of progress of the review by ticking the relevant boxes. Reviews that have progressed beyond the point of completing data extraction at the time of initial registration are not eligible for inclusion in PROSPERO. This field should be updated when any amendments are made to a published record.

The review has not yet started **√**

| Review stage | Started | Completed |
| --- | --- | --- |
| Preliminary searches | No | No |
| Piloting of the study selection process | No | No |
| Formal screening of search results against eligibility criteria | No | No |
| Data extraction | No | No |
| Risk of bias (quality) assessment | No | No |
| Data analysis  Provide any other relevant information about the stage of the review here. | No | No |

# Review team details

## Named contact

The named contact acts as the guarantor for the accuracy of the information presented in the register record. Kimiyo Kikuchi

## Named contact email

Enter the electronic mail address of the named contact. [kimiyo@m.u-tokyo.ac.jp](mailto:kimiyo@m.u-tokyo.ac.jp)

## Named contact address

Enter the full postal address for the named contact. 7-3-1, Hongo, Bunkyo-ku, Tokyo, 113-0033

## Named contact phone number

Enter the telephone number for the named contact, including international dialing code.

+818011461512

## Organisational affiliation of the review

Full title of the organisational affiliations for this review, and website address if available. This field may be completed as 'None' if the review is not affiliated to any organisation.

Department of Community and Global Health, Graduate Schol of Medicine, The University of Tokyo Website address:

## Review team members and their organisational affiliations

Give the title, first name and last name of all members of the team working directly on the review. Give the organisational affiliations of each member of the review team.

| Title | First name | Last name | Affiliation |
| --- | --- | --- | --- |
| Dr | Kimiyo | Kikuchi |  |

## Funding sources/sponsors

Give details of the individuals, organizations, groups or other legal entities who take responsibility for initiating, managing, sponsoring and/or financing the review. Any unique identification numbers assigned to the review by the individuals or bodies listed should be included.

- 1. The Department of Community and Global Health, Graduate School of Medicine, The University of Tokyo. 2. Japan International Cooperation Agency.

## Conflicts of interest

List any conditions that could lead to actual or perceived undue influence on judgements concerning the main topic investigated in the review.

Are there any actual or potential conflicts of interest? None known

## Collaborators

Give the name, affiliation and role of any individuals or organisations who are working on the review but who are not listed as review team members.

Title First name Last name Organisation details

# Review methods

## Review question(s)

State the question(s) to be addressed / review objectives. Please complete a separate box for each question. What is the effectiveness of the continuum of care on maternal and newborn mortality, morbidity, and health behavior?

## Searches

Give details of the sources to be searched, and any restrictions (e.g. language or publication period). The full search strategy is not required, but may be supplied as a link or attachment.

We will conduct literature search from medical databases including: PubMed/MEDLINE, CINAHL, EMBASE, and ISI Web of Knowledge. We will limit the search to 15 years of publication (1999 to 2013). We will also conduct hand search using references of several papers of interest.

## URL to search strategy

If you have one, give the link to your search strategy here. Alternatively you can e-mail this to PROSPERO and we will store and link to it.

I give permission for this file to be made publicly available Yes

## Condition or domain being studied

Give a short description of the disease, condition or healthcare domain being studied. This could include health and wellbeing outcomes.

The maternal, newborn and child health continuum of care has been played up as a potential power to reduce their mortality risk [Kerber 2007, Tinker 2005, WHO 2010].The World Health Organization stated that maternal, newborn and child health interventions could be effective only if they are carried out together, and with home/family and communities [WHO 2005]. The continuum of care is a combination of time and place dimensions cares in accordance with local needs and capacity [Kerber 2007] . As a child’s life depends on mother’s health, continued care access

throughout from pre-pregnancy to childhood is considered as indispensable to the process. Also, high quality and coverage of care for mothers and newborns could be achieved by integrating different service levels including home/family, community, and health facilities. Mothers’ and newborns' health should be assured not by cross-cutting programs approach, but by integrated programs approach [Lawn 2006]. Despite the importance of the continuum of care, most of the previous studies assessed effectiveness of single intervention in cross-cutting approach. Therefore, we aim to conduct this systematic review that may be useful for policy makers and other players in the maternal, newborn and child health field.

## Participants/population

Give summary criteria for the participants or populations being studied by the review. The preferred format includes details of both inclusion and exclusion criteria.

Reproductive age women, newborns and community people. Inclusion criteria: 15-49 years old women, their newborns including unborn children, and community people where they live.

## Intervention(s), exposure(s)

Give full and clear descriptions of the nature of the interventions or the exposures to be reviewed

Continued care interventions given to mothers, newborns and community. The interventions are assessed based on WHO recommended interventions for improving maternal and newborn health [WHO 2007].

## Comparator(s)/control

Where relevant, give details of the alternatives against which the main subject/topic of the review will be compared (e.g. another intervention or a non-exposed control group).

Mothers, newborns and communities that did not receive the intervention explained above.

## Types of study to be included initially

Give details of the study designs to be included in the review. If there are no restrictions on the types of study design eligible for inclusion, this should be stated.

Randomized controlled trials. Quasi experimental design. Longitudinal or cohort studies with a comparison group.

## Context

Give summary details of the setting and other relevant characteristics which help define the inclusion or exclusion criteria.

## Primary outcome(s)

Give the most important outcomes.

- 1. Mortality of mothers or newborns. 2. Stillbirth, disability, and morbidity of mothers or newborns. 3. Health seeking behaviors of mothers or communities.

Give information on timing and effect measures, as appropriate.

## Secondary outcomes

List any additional outcomes that will be addressed. If there are no secondary outcomes enter None.

Other maternal and newborn related health outcomes that may be common among the literature reviewed including community people's behavior and perception.

Give information on timing and effect measures, as appropriate.

## Data extraction, (selection and coding)

Give the procedure for selecting studies for the review and extracting data, including the number of researchers involved and how discrepancies will be resolved. List the data to be extracted.

## Risk of bias (quality) assessment

State whether and how risk of bias will be assessed, how the quality of individual studies will be assessed, and whether and how this will influence the planned synthesis.

We will assess risk of bias (RoB) Individual studies selected will be analyzed for: 1. Selection bias 2. Performance bias 3. Detection bias 4. Attrition bias 5. reporting bias

## Strategy for data synthesis

Give the planned general approach to be used, for example whether the data to be used will be aggregate or at the level of individual participants, and whether a quantitative or narrative (descriptive) synthesis is planned. Where

appropriate a brief outline of analytic approach should be given.

We will run descriptive analysis of individual studies according to the intervention, its study design, participants, country, duration, outcome, quality, and risk of bias. Based on the nature of outcome variable reported, we will run analyses to examine the effectiveness of intervention on maternal and newborn outcomes mentioned earlier. If we have enough RCTs with quality data reported, we will conduct meta analyses to find the effectiveness of intervention. In event of lacking of similar data on outcome variable, we will run a narrative summary.

## Analysis of subgroups or subsets

Give any planned exploration of subgroups or subsets within the review. ‘None planned’ is a valid response if no subgroup analyses are planned.

None planned

# Review general information

## Type of review

Select the type of review from the drop down list. Intervention

## Language

Select the language(s) in which the review is being written and will be made available, from the drop down list. Use the control key to select more than one language.

English

Will a summary/abstract be made available in English? Yes

## Country

Select the country in which the review is being carried out from the drop down list. For multi-national collaborations select all the countries involved. Use the control key to select more than one country.

Ghana, Japan

## Other registration details

List places where the systematic review title or protocol is registered (such as with he Campbell Collaboration, or The Joanna Briggs Institute). The name of the organisation and any unique identification number assigned to the review by that organization should be included.

## Reference and/or URL for published protocol

Give the citation for the published protocol, if there is one.

Give the link to the published protocol, if there is one. This may be to an external site or to a protocol deposited with CRD in pdf format.

I give permission for this file to be made publicly available No

## Dissemination plans

Give brief details of plans for communicating essential messages from the review to the appropriate audiences. Publication in a scientific journal.

Do you intend to publish the review on completion? Yes

## Keywords

Give words or phrases that best describe the review. (One word per box, create a new box for each term) continuum of care

maternal mortality neonatal mortality

## Details of any existing review of the same topic by the same authors

Give details of earlier versions of the systematic review if an update of an existing review is being registered, including full bibliographic reference if possible.

## Current review status

Review status should be updated when the review is completed and when it is published. Ongoing

## Any additional information

Provide any further information the review team consider relevant to the registration of the review.

## Details of final report/publication(s)

This field should be left empty until details of the completed review are available. Give the full citation for the final report or publication of the systematic review.

Give the URL where available.
