# Supplementary material for: Effective Linkages of Continuum of Care for Improving Neonatal, Perinatal, and Maternal Mortality: A Systematic Review and Meta-Analysis
Source: PLoS One. 2015 Sep 30;10(9):e0139288. doi: 10.1371/journal.pone.0139288 (PMC4589290; doi:10.1371/journal.pone.0139288)
Supplement: S2 Text — (DOCX) [file pone.0139288.s003.docx]

**S2 Text**

**Search Terms for “Effective Linkages of Continuum of Care at Improving Neonatal, Perinatal, and Maternal Mortality” Review – Published Literature**

**Research Question**

**Which linkages of CoC components reduce maternal, neonatal, or perinatal mortality in low- and middle- income countries?**

***Strategy 1: Search terms for “continuum of care”***

***Search terms of “antenatal care”***

"prenatal care"[MeSH Terms] OR ("prenatal"[All Fields] AND "care"[All Fields]) OR "prenatal care"[All Fields] OR ("antenatal"[All Fields] AND "care"[All Fields]) OR "antenatal care"[All Fields]

***Search terms of “delivery”***

(skilled[All Fields] AND ("parturition"[MeSH Terms] OR "parturition"[All Fields] OR "birth"[All Fields]) AND attendant[All Fields]) OR (facility[All Fields] AND ("delivery, obstetric"[MeSH Terms] OR ("delivery"[All Fields] AND "obstetric"[All Fields]) OR "obstetric delivery"[All Fields] OR "delivery"[All Fields]))

***Search terms of “postnatal care”***

(postnatal care[MeSH Terms] OR ("postnatal"[All Fields] AND "care"[All Fields]) OR "postnatal care"[All Fields])

***Strategy 2: Combination of search terms for “pregnant woman/mother” & “neonate/newborn” & “mortality” terms***

***Search terms of “pregnant woman/mother”***

("pregnant women"[MeSH Terms] OR ("pregnant"[All Fields] AND "women"[All Fields]) OR "pregnant women"[All Fields]) OR ("mothers"[MeSH Terms] OR "mothers"[All Fields]) OR ("women"[MeSH Terms] OR "women"[All Fields])

***Search terms of “neonate/newborn”***

("infant, newborn"[MeSH Terms] OR ("infant"[All Fields] AND "newborn"[All Fields]) OR "newborn infant"[All Fields] OR "newborn"[All Fields]) OR ("infant"[MeSH Terms] OR "infant"[All Fields]) OR (“neonate”[All Fields]))

***Search terms of “mortality”***

(“maternal mortality"[MeSH Terms] OR ("maternal"[All Fields] AND "mortality"[All Fields]) OR "maternal mortality"[All Fields]) OR ("perinatal mortality"[MeSH Terms] OR ("perinatal"[All Fields] AND "mortality"[All Fields]) OR "perinatal mortality"[All Fields]) OR ("infant mortality"[MeSH Terms] OR ("infant"[All Fields] AND "mortality"[All Fields]) OR "infant mortality"[All Fields] OR ("neonatal"[All Fields] AND "mortality"[All Fields]) OR "neonatal mortality"[All Fields])

***Strategy 3: Combination of “continuum of care” & “pregnant woman/mother” & “neonate/newborn” & “mortality” terms***

***Combination search terms of “antenatal care”***

("prenatal care"[MeSH Terms] OR ("prenatal"[All Fields] AND "care"[All Fields]) OR "prenatal care"[All Fields] OR ("antenatal"[All Fields] AND "care"[All Fields]) OR "antenatal care"[All Fields]) AND (("pregnant women"[MeSH Terms] OR ("pregnant"[All Fields] AND "women"[All Fields]) OR "pregnant women"[All Fields]) OR ("mothers"[MeSH Terms] OR "mothers"[All Fields]) OR ("women"[MeSH Terms] OR "women"[All Fields])) AND (("infant, newborn"[MeSH Terms] OR ("infant"[All Fields] AND "newborn"[All Fields]) OR "newborn infant"[All Fields] OR "newborn"[All Fields]) OR ("infant"[MeSH Terms] OR "infant"[All Fields]) OR (“neonate”[All Fields]))) AND ((“maternal mortality"[MeSH Terms] OR ("maternal"[All Fields] AND "mortality"[All Fields]) OR "maternal mortality"[All Fields]) OR ("perinatal mortality"[MeSH Terms] OR ("perinatal"[All Fields] AND "mortality"[All Fields]) OR "perinatal mortality"[All Fields]) OR ("infant mortality"[MeSH Terms] OR ("infant"[All Fields] AND "mortality"[All Fields]) OR "infant mortality"[All Fields] OR ("neonatal"[All Fields] AND "mortality"[All Fields]) OR "neonatal mortality"[All Fields]))

***Combination search terms of “delivery”***

((skilled[All Fields] AND ("parturition"[MeSH Terms] OR "parturition"[All Fields] OR "birth"[All Fields]) AND attendant[All Fields]) OR (facility[All Fields] AND ("delivery, obstetric"[MeSH Terms] OR ("delivery"[All Fields] AND "obstetric"[All Fields]) OR "obstetric delivery"[All Fields] OR "delivery"[All Fields]))) AND (("pregnant women"[MeSH Terms] OR ("pregnant"[All Fields] AND "women"[All Fields]) OR "pregnant women"[All Fields]) OR ("mothers"[MeSH Terms] OR "mothers"[All Fields]) OR ("women"[MeSH Terms] OR "women"[All Fields])) AND (("infant, newborn"[MeSH Terms] OR ("infant"[All Fields] AND "newborn"[All Fields]) OR "newborn infant"[All Fields] OR "newborn"[All Fields]) OR ("infant"[MeSH Terms] OR "infant"[All Fields]) OR (“neonate”[All Fields]))) AND ((“maternal mortality"[MeSH Terms] OR ("maternal"[All Fields] AND "mortality"[All Fields]) OR "maternal mortality"[All Fields]) OR ("perinatal mortality"[MeSH Terms] OR ("perinatal"[All Fields] AND "mortality"[All Fields]) OR "perinatal mortality"[All Fields]) OR ("infant mortality"[MeSH Terms] OR ("infant"[All Fields] AND "mortality"[All Fields]) OR "infant mortality"[All Fields] OR ("neonatal"[All Fields] AND "mortality"[All Fields]) OR "neonatal mortality"[All Fields]))

***Combination search terms of “postnatal care”***

((postnatal care[MeSH Terms] OR ("postnatal"[All Fields] AND "care"[All Fields]) OR "postnatal care"[All Fields])) AND (("pregnant women"[MeSH Terms] OR ("pregnant"[All Fields] AND "women"[All Fields]) OR "pregnant women"[All Fields]) OR ("mothers"[MeSH Terms] OR "mothers"[All Fields]) OR ("women"[MeSH Terms] OR "women"[All Fields])) AND (("infant, newborn"[MeSH Terms] OR ("infant"[All Fields] AND "newborn"[All Fields]) OR "newborn infant"[All Fields] OR "newborn"[All Fields]) OR ("infant"[MeSH Terms] OR "infant"[All Fields]) OR (“neonate”[All Fields]))) AND ((“maternal mortality"[MeSH Terms] OR ("maternal"[All Fields] AND "mortality"[All Fields]) OR "maternal mortality"[All Fields]) OR ("perinatal mortality"[MeSH Terms] OR ("perinatal"[All Fields] AND "mortality"[All Fields]) OR "perinatal mortality"[All Fields]) OR ("infant mortality"[MeSH Terms] OR ("infant"[All Fields] AND "mortality"[All Fields]) OR "infant mortality"[All Fields] OR ("neonatal"[All Fields] AND "mortality"[All Fields]) OR "neonatal mortality"[All Fields]))
